# Is there any effect of coenzyme Q10 on prevention of myringosclerosis? Experimental study with rats

**DOI:** 10.5935/1808-8694.20130053

**Published:** 2015-10-04

**Authors:** Filiz Aydoğan, Emine Aydın, Eren Taştan, Şükran Akgedik, Ahmet Tekeli, Hüseyin Üstün

**Affiliations:** a Medical Doctor (Department of Otorhinolaryngology, Ankara Training and Research Hospital).; b Medical Doctor (Department of Pathology, Ankara Training and Research Hospital).; c Assist. Prof. Dr. (Department of Animal Science Feeds and Animal Nutrition, Faculty of Agriculture Yuzuncu Yil University). Institution Department of Otorhinolaryngology, Ankara Training and Research Hospital.

**Keywords:** antioxidants, sclerosis, tympanic membrane

## Abstract

Recent studies have shown that the formation of myringosclerosis could be reduced by the application of antioxidant enzymes and elements.

**Objective:**

The aim of this study was to investigate the effectiveness of coenzyme Q10 on the prevention of experimentally induced myringosclerosis.

**Method:**

Forty-eight healthy female wistar albino rats were bilaterally myringotomized and divided into four groups randomly. Group A received no treatment, group B was administered oral coenzyme Q10. Group C was treated with topical saline solution, group D received topically coenzyme Q10. On the 15^th^ day of treatment, tympanic membranes were examined by otomicroscopy. Myringosclerotic lesions were documented semiquantitatively by using 4-point scale. After harvesting tympanic membranes were evaluated histopathologically.

**Results:**

In group D (topical coenzyme Q10), we observed otitis within the first four days of the study and this group was excluded from the study. Regarding otomicroscopic examinations, there were no significant differences among groups in myringosclerosis formation (*p* = 0.241). When group A (non treatment) compared to groups B and C regarding histopathologic examination, the results demonstrated statistical significant differences (*p* = 0.004; *p* < 0.001), respectively. There was no statisticaly significant difference between groups B and C (*p* = 0.160).

**Conclusion:**

Oral administration of coenzyme Q10 did not reduce myringosclerosis formation in myringotomized rats.

## INTRODUCTION

Tympanosclerosis is a degenerative process in the connective tissue layer and effects both the tympanic membrane (TM) and the middle ear mucosa^1^, ^2^. The most common localization of tympanosclerosis is in the TM and called myringosclerosis (MS)^1^. It is hyalinization and calcification of the collagen layer in certain areas of the TM^3^, ^4^, ^5^, ^6^. Histologically, there is an increase in collagen fibers due to progressive fibroblast infiltration as well as hyaline degeneration and extracellular calcium deposition within lamina propria^2^, ^3^, ^4^. It appears as semicircular crescent or horseshoe-shaped white chalky patches usually at the anterior and posterior inferior quadrants of the TM^6^. Myringosclerosis (MS) is a common sequele of recurrent otitis media, otitis media with effusion, chronic otitis media and ventilation tube insertion^3^, ^4^, ^5^. Although the morphology of MS has been well described, the exact etiology and pathogenesis is still inadequately understood. Several hypotheses have been proposed for the etiology of MS including: reactive oxygen species, immunologic sensitivity, mechanical injury, metabolic disturbance and inflammatory reaction^3^, ^7^, ^8^, ^9^.

Coenzyme Q10 (CoQ10) is regenerable, bioenergetic, powerful, intracellular antioxidant^10^, ^11^. It exists in all cells and is endogenously synthesized as well as food intake^11^, ^12^. It acts as an electron and proton carrier in the mitochondrial respiratory chain and is necessary for ATP production^10^. It protects phospholipids and mitochondrial membrane proteins from peroxidation and protects DNA against the oxidative damage that accompanies lipid peroxidation^11^. CoQ10 functions as an antioxidant, supporting the regeneration of other antioxidant; influencing the stability, fludity and permeability of membranes; and stimulating cell growth and inhibiting cell death^12^. It is present in the body in both the reduced (ubiquinol) and oxidised (ubiquinone) forms^13^. Its reduced form, ubiquinol is also an antioxidant^10^. When cell membranes are oxidized, ubiquinol is the first antioxidant consumed^14^. Moreover, the formation of oxidized lipids and the consumption of α-tocopherol are suppressed while ubiquinol is present^14^.

Recent studies have shown that the formation of MS, after experimental myringotomy, could be reduced by the application of antioxidant enzymes and elements^1^, ^3^. In the light of these studies, we also thought that MS might be reduced or prevented by the application of the anti-oxidant CoQ10.

The aim of this study was to investigate possible preventive effect of CoQ10 on the development of MS in the TM of myringotomized rats using otomicroscopy and histopathology. To our knowledge, this is the first study to evaluate the preventive effect of topical and oral administration of CoQ10 on the development of MS.

## METHOD

### Experimental Design

The experimental design was approved by the Ethics Research Committee in Animal Experiments (prot. nº: 31/03/2010-60/308), and this study was conducted in compliance with the guidelines for animal experimentation at the department of Laboratory Animal Science of Medical School. All animals care and procedures were performed humanely. The animals were kept in ordinary cages with free access to food and water and at a temperature of 20 to 22°C with artificial lighting in a period of 12 hours. They were given pellets (2700 ME Kcal/kg, 23% HP) and water ad libitum and used after 1 week of quarantine and acclimatization.

Forty-eight healthy female wistar albino rats, mean weight 220 g, were used in this study. The animals that had external or middle ear infection and adhesion, perforation, or retraction in the TM during otomicroscopic examination were excluded from the study. They were anesthetized by ketamine hydrocloride (50 mg/kg, intramuscular). Myringotomies were performed bilaterally in the posterior superior quadrant of TMs through an ear speculum with sterile pic under the otomicroscope (Opmi 1, Zeiss, Germany). No intratympanic bleeding was observed during this procedure.

The rats were separated into four groups randomly (groups A, B, C, D). Each group included 12 rats. Group A served as the control group and were not administered treatment. In group B, animals received 100 mg/kg/day CoQ10 with feeding needle and group C received 0.1 ml saline solution topically in both ears. Group D received 100 mg/kg/day CoQ10 (GNC 50 mg soft gelatine capsul diluted with 0.2 ml saline) topically in both ears. Saline solution and CoQ10 diluted with saline were dropped into external ear canal using a syringe. The medicine was administered daily for 15 days. The dose of the CoQ10 supplement was determined according to the literature^14^. Two TMs of another rat were served for histopathologic comparison between the TMs of study groups and the healthy intact TM^7^. TMs of anesthetized rats were examined by otomicroscopy on the 15^th^ day after treatment. The status of TMs was evaluated and myringosclerotic lesions were documented semiquantitatively by using 4-point scale: (0), no visible myringosclerotic plaques; (1), occasional MS with white halo around umbo; (2), moderate MS with white halo around umbo and white line beside the handle of the malleus and along the annulus; and (3), severe MS with confluent whitish deposits, forming a horseshoe shaped pattern^15^.

### Histopathologic Evaluation

On the 15^th^ day, the rats were sacrificed painlessly by high dose pentobarbital (80 mg/kg, intraperitoneal injection). TM and surrounding bony annulus were removed together. They were fixed with 10 percent formaldehyde solution and decalcified with 7 percent nitric oxide solution. The specimens were embedded paraffin. After routine processing, the specimens were sectioned on 4 µm thick. Sections were prepared at the level of pars tensa where myringotomies were performed and stained with hematoxylin-eosin and toluidine blue. Toluidine blue staining was used to evaluate the sclerotic changes in the connective tissue of lamina propria. In the light microscope, stained specimens were evaluated by blinded pathologist according to degree of sclerosis, hyalinization and intensity of fibroblastic proliferation in the lamina propria and thickness of TM. They were documented by comparing TM of normal rat and using 5 point scale from 0 (normal) to 4. The groups were compared according to findings of otomicroscopic and histopathologic examinations.

### Statistical Analysis

Statistical analysis was performed by Statistical Package for Social Sciences (SPSS) 11.5 software (SPSS Inc., Chicago, IL, United States). Data were expressed as median (minimum-maximum) for ordinal data and number of cases and percentages for nominal variables. The median differences among group were analyzed by Kruskal Wallis test. When the p-value from the Kruskal Wallis test are statistically significant to know which group differ from which others by using Conover's multiple comparison test were used. Nominal data were evaluated by Pearson Chi-square or Fisher's exact test, where appropriate. A *p* value less than 0.05 was considered statistically significant.

## RESULTS

### Excluded group because of otitis and death

Two rats (1/B, 1/C) died because of natural causes at the end of the study. In group D (topical), we observed otitis within the first four days of the study and this group was excluded from the study. In otomicroscopic examination because of otitis media, 5 ears (2/A, 1/B, 2/C) and in histopathologic examination 1 additional ear (1/C) were excluded from the study. Consequently, total 6 ears and group D (topical) were excluded from the study because of otitis.

### Otomicroscopic examination

All TM perforations in four groups were healed. Table 1 summarizes the results of otomicroscopic examination. When statistical analysis was evaluated according to otomicroscopic examinations, there were no significant differences among groups in MS formation (*p* = 0.241). According to otomicroscopic and histopathologic evaluations, comparison of median values among groups is shown in Table 2.

**Table 1:** Otomicroscopic examination of sclerotic lesions.

Groups	Ears	Myringosclerosis scale (point)			
		0	1	2	3
A (non treatment)	22	3	8	11	0
B (oral CoQ10)	21	1	6	14	0
C (saline)	19	3	3	9	4

CoQ10: Coenzyme Q10.

**Table 2:** Comparison of median values in otomicroscopic and histopathologic examinations.

Groups and ears	Otomicroscopic	Histopathologic
A (non treatment) 22	1.5 (0-2)	1 (0-4)
B (oral CoQ10) 21	2 (0-2)	2 (0-4)
C (saline) 19	2 (0-3)	3 (1-4)
*p* value	0.241	< 0.001

Data were expressed as median (minimum-maximum); CoQ10: Coenzyme Q10.

### Histopathologic examination

**Figure 1:**
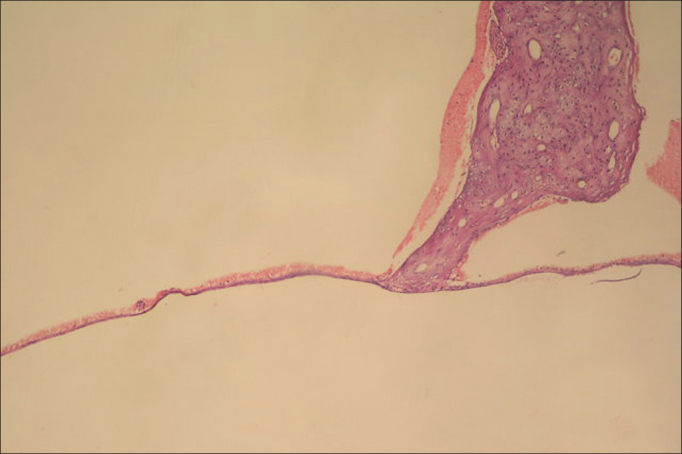
Appearence of normal tympanic membrane (0 point scale) in group A (hematoxylin and eosin, original magnification x100).

**Figure 2:**
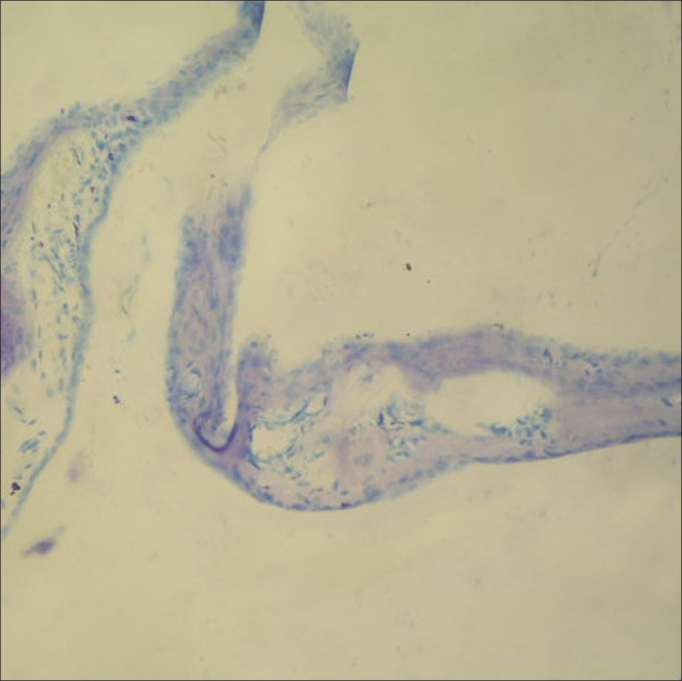
In tympanic membrane evaluated as 4 point scale from group B, the fibroblast proliferation, markedly increased of membrane thickness, sclerotic changes are shown (toluidine blue, original magnification x200).

**Figure 3:**
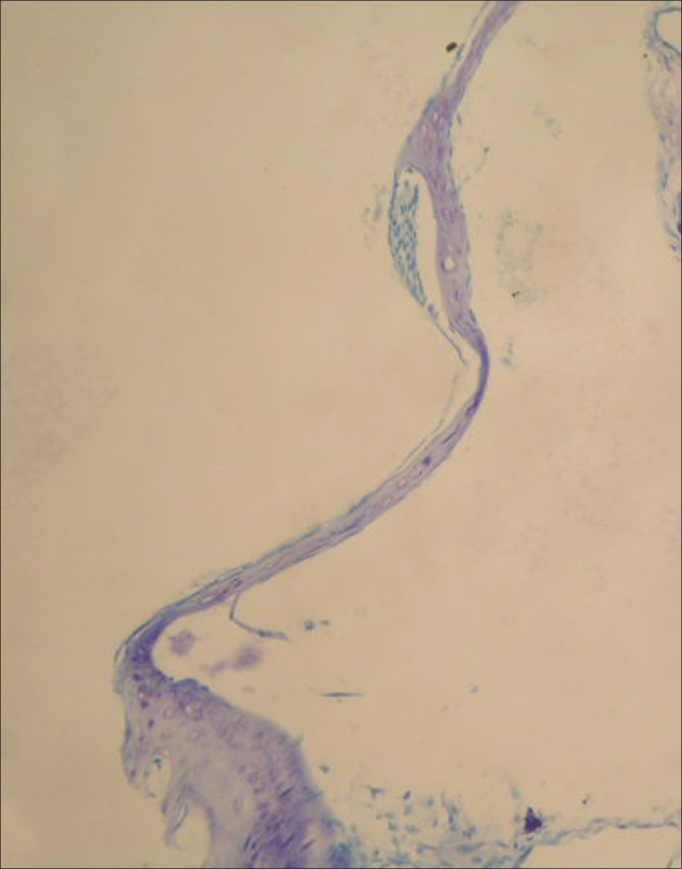
In tympanic membrane evaluated as 2 point scale from group C, moderately thickened and sclerotic plaque are shown (toluidine blue, original magnification x200).

Normal TMs, were thin and free of inflammatory cells (Figure 1). There were extensive sclerotic lesions and intensity of fibroblasts located in the lamina propria and increased thickness of the TM in groups B (oral), C (saline) (Figures 2, 3). The TMs from group A (non treatment) were thinner than those from groups B (oral), C (saline) and the fibroblastic activity in lamina propria and sclerotic lesions were also less pronounced in this group. According to groups the findings of histopathologic examination were shown in Table 3. When group A (non treatment) compared to groups B (oral) and C (saline) regarding histopathologic examination, the results demonstrated statistical significant differences (*p* = 0.004; *p* < 0.001), respectively. There was no statisticaly significant difference between groups B (oral) and C (saline) (*p* = 0.160).

**Table 3:** The findings of histopathologic examination of TMs.

Groups	Ears	Histopathologic Scale				
		0	1	2	3	4
A (non treatment)	22	1	12	6	2	1
B (oral CoQ10)	21	3	0	8	8	2
C (saline)	19	0	2	4	10	3

TMs: Tympanic membranes; CoQ10: Coenzyme Q10.

## DISCUSSION

Recent studies emphasized that oxygen-derived free radicals, mechanical injury and an inflammatory disease may be the main factors in the formation of MS^8^, ^9^. Mattsson et al.^9^ observed that MS lesions were shown to increase in number with increased oxygen concentrations when myringotomized rats were exposed to different oxygen concentrations and suggested that the mechanism involved could be related to the formation of oxygen radicals. The myringotomy permits passage of ambient air into the middle ear cavity resulting in a relatively hyperoxic condition. This condition would lead to an increased oxygenation of the tissues, and could provoke an increased production of oxygen-derived free radicals, which may trigger irreversible tissue damage involving fibrosis, hyaline degeneration as seen in MS^9^.

There have been many experimental studies attempting to prevent the development of MS by applying free radical scavengers topically or systematically^3^, ^4^, ^5^, ^7^, ^15^, ^16^. It was reported that topically applied free radical scavengers such as ascorbic acid^15^, N-acetylcysteine^16^, copper zincsuperoxide dismutase plus catalase and deferoxamine inhibited the development of MS^6^. On the other hand, previous studies also noticed that systematically administered L-carnitine^17^, selenium^5^, caffeic acid phenethyl ester^3^, vitamin E (alpha-tocopherol)^4^, gingko biloba^7^ were effective to prevent MS formation. In addition to, Polat et al.^1^ demonstrated that both topical and intramuscular application of vitamin E reduces the formation of MS and the reactive oxygen species in TMs. Consequently, the findings of reports have supported the hypothesis that the formation of reactive oxygen species contributed significantly to the development of MS^1^, ^15^.

It was found that the development of MS after myringotomy can be arrested by antiinflammatory drugs such as the dexametason or topical fenspiride.^8^ Some agents reduced the development of MS have both antioxidant and anti-inflammatory effects^3^, ^7^, ^16^.

In group D (topical), we observed otitis although previous animal studies did not report that topically applied agents caused otitis and/or had irritative effect in TM^1^, ^15^, ^16^. Possible chemical irritative effect of topically applied CoQ10 might cause inflammation in the TMs. We thought that topical CoQ10 application had possible chemical irritative effect in TM.

Mattsson et al.^8^ reported that the observation of MS in the otomicroscope did not completely corroborate the findings in the light microscope.

Though many agents are proven to prevent the development of MS in experimental studies, there are no standardized objective findings in the histopathologic evaluation of MS^2^. We also used 5 point scale from (0) to (4) by evaluating degree of sclerosis, hyalinization, intensity of fibroblastic proliferation and thickness of TM. In some earlier studies, the agents that reduced the development of MS led to unwanted thickening of the TM due to an increased number of fibroblasts in histopathologic evaluation^5^, ^15^, ^16^. Contrary to these studies, some authors observed that there was a positive relationship between thickness of TM and degree of MS^2^, ^3^, ^4^, ^7^, ^17^. Our results were also similar to these literature findings.

CoQ10 is a relatively large, hydrophobic molecule. Therefore, absorption of CoQ10 into tissues is often slow and limited^12^. Tissues with high energy requirements, such as the heart, kidney, liver and skeletal muscle, contain high amounts of CoQ10^10^. It was suggested that there were possible discrepancies between extracellular and intracellular CoQ10 concentrations^11^.

## CONCLUSION

In this investigation, it was found that sistemic CoQ10 administration for 15 days to myringotomized rats were not effective to prevent the development of MS. In this context, inefficacy due to dose and duration of applied CoQ10 may be reviewed. Although we could not measure plasma levels of CoQ10, we confirmed that dose and duration of administered CoQ10 in this study provided efficient antioxidant effect regarding to these literature^11^, ^12^, ^18^, ^19^.

Concerning ineffectiveness of CoQ10 on MS, we have speculated that CoQ10 would have unclear mechanism on the development of MS.
